# Effects of post‐exercise glucose ingestion at different solution temperatures on glycogen repletion in mice

**DOI:** 10.14814/phy2.15041

**Published:** 2021-09-23

**Authors:** Yutaka Matsunaga, Sho Koyama, Kenya Takahashi, Yumiko Takahashi, Terunaga Shinya, Hiroki Yoshida, Hideo Hatta

**Affiliations:** ^1^ Department of Sports Sciences The University of Tokyo Tokyo Japan

**Keywords:** glycogen recovery, liver, skeletal muscle, solution temperature

## Abstract

Carbohydrate ingestion is essential for glycogen recovery after exercise. Although studies have investigated methods for enhancement of glycogen repletion with regard to nutrients and their amounts, no studies have examined the effect of temperature of the ingested solution on glycogen recovery. Therefore, this study aimed to investigate the effect of the temperature of glucose solution ingested after exercise on glycogen recovery. Seven‐week‐old male ICR mice were fasted for 16 h and subjected to treadmill running exercise (20 m/min for 60 min) to decrease glycogen storage. Then, the mice were administered glucose (1.5 mg/g body weight) at three different solution temperatures: 4°C, cold solution group (Cold); 37°C, mild solution group (Mild); and 55°C, hot solution group (Hot). Our results revealed that blood glucose, plasma insulin, and muscle glycogen concentrations did not differ among the three groups. In contrast, liver glycogen concentration in the Hot group was significantly higher than that in the post‐exercise and Cold groups (*p* < 0.05). Furthermore, portal glucose concentration was significantly higher in the Hot group than in the Cold group (*p* < 0.01). These observations suggest that postexercise muscle glycogen repletion occurs regardless of glucose solution temperature, and that ingesting hot glucose solution after exercise can be an effective means for liver glycogen repletion compared with cold glucose solution ingestion.

## Graphical abstract

This study examined the effects of different solution temperatures of post‐exercise glucose ingestion on glycogen recovery. At 30 min of recovery, muscle glycogen repletion occurred regardless of glucose solution temperature. Liver glycogen recovery was observed after hot glucose solution ingestion, but not after cold glucose solution ingestion. These observations suggest that ingesting hot glucose solution after exercise can be an effective means for liver glycogen repletion.

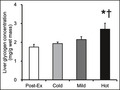

## 1 INTRODUCTION

Exercise‐induced fatigue is caused by complex interactions between various central and peripheral factors. Many muscle properties change during fatigue, including action potentials, extracellular and intracellular ions, muscle glycogen, and many intracellular metabolites (Allen et al., 2008). In addition, fatigue can result from increased body temperature, dehydration, and decreased blood glucose levels (Ament & Verkerke, 2009). Among the fatigue factors, glycogen as an energy source plays a major role. Muscle glycogen utilization increase as exercise intensity progresses (van Loon et al., 2001; Romijn et al., 1993). Glycogen content is related to endurance exercise (Bergström et al., 1967) and intermittent sprint exercise performance (Skein et al., 2012). Enhancing glycogen resynthesis after exercise affects subsequent exercise performance (Alghannam et al., 2016; Casey et al., 2000; Williams et al., 2003). For instance, Alghannam et al. (2016) reported that high carbohydrate drink ingestion enhanced glycogen repletion by 73% and subsequent endurance exercise duration by 66% compared to low carbohydrate drink ingestion. Therefore, it is crucial to recover glycogen quickly and efficiently for physically active people and athletes who exercise multiple times a day.

Carbohydrate ingestion enhances skeletal muscle glycogen recovery after exercise (Pascoe et al., 1993). Glycogen synthesis is maximal at a carbohydrate concentration of approximately 1.2 g/kg/h (Jentjens & Jeukendrup, 2003), ingested immediately after exercise (Ivy et al., 1988). In addition, it has been reported that the combination of carbohydrates with other nutrients, such as proteins, increases insulin secretion and enhances glycogen recovery (Zawadzki et al., 1992). Therefore, several studies have focused on methods to enhance glycogen recovery in terms of timing and amount of glucose ingestion as well as additional nutrients ingested with glucose.

Gonzalez et al. (2017) posited that rapidly digested and absorbed carbohydrates might accelerate glycogen repletion. One of the factors that affect the digestion and absorption is the temperature of the ingested solution. For instance, Costill and Saltin (1974) reported that a cold solution (5°C) of glucose with NaCl accelerated gastric emptying compared with a warm solution (35°C). Hence, it is possible that the ingestion of cold solution enhances glycogen recovery by delivering glucose to the tissues rapidly. In contrast, however, Sun et al. (1988) reported that a cold drink (4°C) resulted in slower gastric emptying than warm drinks (37°C). In another report, the frequency of gastric contractions after water intake was lower in a cold (2°C) trial than in a hot (60°C) trial (Fujihira et al., 2020). Thus, conflicting and inconsistent results have been reported. Furthermore, although previous studies have shown that differences in solution temperature affect gastric movements and gastric emptying, it is unclear whether a difference in solution temperature affects absorption in the digestive tract or glycogen recovery in the muscle and liver. Therefore, the purpose of this study was to clarify the effects of the temperature of ingested glucose solutions on digestion, absorption, and glycogen recovery after exercise.

## 2 MATERIALS AND METHODS

### 2.1 Ethical approval

In this study, all procedures were performed in accordance with the ethical standards of the Committee on Animal Care and Use of The University of Tokyo. All experimental protocols were approved by the Animal Experimental Committee of The University of Tokyo (no. 30‐6).

### 2.2 Experimental animals

Six‐week‐old male ICR mice were obtained from CLEA Japan Inc. All mice were housed individually in an environment maintained at 25°C with a 12/12‐h light‐dark cycle (light: 19:00–7:00, dark: 7:00–19:00) and were provided with water and standard chow (3.59 kcal/g; 55.3% carbohydrates, 23.1% protein, 5.1% fat, 5.8% ash, 2.8% fiber, and 7.9% moisture; MF diet; Oriental Yeast Co., Ltd.) ad libitum. They were acclimated to the environment for 1 week and were familiarized with the treadmill running exercise at a speed of 10–20 m/min for 10 min for 3 days before starting the experiment.

### 2.3 Materials

D‐Glucose (047‐31161; Fujifilm Wako Chemical Corporation) was diluted with water up to a concentration of 7.5%.

### 2.4 Experimental protocols

Unless otherwise noted, all procedures were performed while animals were fully conscious. After 16 h of fasting, the mice were subjected to treadmill running exercise (20 m/min for 60 min) to decrease the glycogen content in skeletal muscle and liver. Additionally, since this study focused on digestion and absorption, it was necessary to empty the digestive tract before oral glucose administration. Therefore, we set a fasting period of 16 h. The mice were then randomly divided into three groups: 4°C, cold solution group (Cold, *n* = 8); 37°C, mild solution group (Mild, *n* = 8); and 55°C, hot solution group (Hot, *n* = 8). The glucose solution (1.5 mg/g body weight [BW]) was administered via oral gavage. Rectal temperature measurement and tail vein blood sampling were performed at 0, 15, and 30 min after glucose ingestion. Animals were retained in a cloth bag for 2 min for the duration of the measurement and sampling. Blood samples were then centrifuged (4°C, 5000 *g*, 10 min), and the plasma fractions were rapidly frozen in liquid nitrogen and stored at –80°C until further analysis.

Given that both rectal temperature measurement and tail vein blood sampling could interfere with glycogen recovery, we performed the same experiment separately. After a 1 week washout period, the animals in all the groups were treated similarly to the first experiment. Mice were anesthetized using isoflurane and euthanized by blood collection from the portal vein and inferior vena cava 30 min after the postexercise glucose ingestion. Because we expected that digestion and absorption would affect early phase of postexercise glycogen recovery, we set this time frame. After collecting 50 μL of blood from the portal vein, the remaining blood samples were collected from the inferior vena cava. In addition to the soleus, plantaris, tibialis anterior, and gastrocnemius muscles, the liver and stomach were harvested. Muscle and liver samples were rapidly frozen in liquid nitrogen and stored at –80°C until further analysis. As reference markers, we used a control group (Con, *n* = 6) in which tissue was harvested from sedentary animals and a postexercise group (Post‐Ex, *n* = 6) in which tissue was harvested immediately after exercise.

### 2.5 Analyses

#### 2.5.1 Rectal temperature

Rectal temperature was measured as described previously (Meyer et al., 2017). Mice were hand‐restrained and their tails were lifted. A probe was gently inserted into the rectum to a depth of 1 cm and held for approximately 5 s. Rectal temperature was measured using an animal thermometer (KN‐91‐AD1687; Natsume Seisakusho Co. Ltd.).

#### 2.5.2 Blood and plasma substrate concentrations

The tail vein and portal blood glucose concentrations were measured using an auto analyzer (GT‐1840; GLUCOCARD Plus Care, Arkray Inc.). Plasma insulin concentration was measured using the Morinaga Ultra‐Sensitive Mouse Insulin ELISA Kit (M1104, Morinaga Institute of Biological Science, Inc.).

#### 2.5.3 Intragastric glucose concentration

After gastrectomy, the gastric contents were washed with 60 μL H_2_O/g BW of water. The water was collected and centrifuged at 2000 *g* for 10 min at 4°C. Glucose concentration in the supernatants was measured using the Glucose CII Test Wako Kit (439‐90901; Fujifilm Wako Chemical Corporation).

#### 2.5.4 Muscle and liver glycogen concentrations

The glycogen content of the muscles and liver was measured using the phenol‐sulfuric acid method, as described previously (Lo et al., 1970). Liver fragments (approximately 10 mg) and whole muscles were weighed, added to 300 μL of 30% (w/v) KOH saturated with Na_2_SO_4_, and heated at 98°C until completely dissolved. The resulting solutions were mixed with 360 μL ethanol and placed on ice for 30 min, followed by centrifugation (4°C, 5000 *g*, 15 min). Next, the supernatants were removed and the glycogen‐containing precipitate was dissolved in distilled water. Subsequently, 5% (v/v) phenol and sulfuric acid were added to the solution, and the mixture was allowed to react for 15 min before absorbance was measured at 490 nm.

#### 2.5.5 Tissue metabolites

Liver glucose and glucose‐6‐phosphate (G‐6‐P) concentrations were measured using enzymatic colorimetric methods, as described previously (Takahashi et al., 2020; Zhu et al., 2011). Briefly, the liver was homogenized in 0.6 N HClO_4_ buffer using a tissue crusher (μT‐01; TAITEC). After centrifugation (4°C, 12,000 *g*, 10 min), the supernatants were neutralized with 1 M NaOH. Thirty microliters of neutralized samples were added to 70 μL of either glucose assay solution (20 μM MgCl_2_, 25 μM NADP+, 0.5 mM WST‐1, 10 μM 1‐mPMS, 0.2 U glucose dehydrogenase, and 50 mM Tris‐HCl; pH 8.5) or G‐6‐P assay solution (20 μM MgCl_2_, 25 μM NADP+, 0.5 mM WST‐1, 10 μM 1‐mPMS, 0.2 U G‐6‐P dehydrogenase, and 50 mM Tris‐HCl; pH 8.5). The absorbance at 440 nm was measured after incubation for 30 min at room temperature (26°C) in the dark.

#### 2.5.6 Western blot analysis

The liver was homogenized using the radioimmunoprecipitation assay lysis buffer (20–188; Millipore) containing a protease inhibitor (1183617001, Complete Mini EDTA‐free; Roche Life Science) and phosphatase inhibitor (04906837001, PhosSTOP phosphatase inhibitor cocktail; Roche Life Science). The homogenates were placed on ice for 60 min and centrifuged (4°C, 1500 *g*, 20 min). The total protein content of the samples was determined using a BCA protein assay kit (23227; Pierce). Proteins (10 μg of each sample) separated by sodium dodecyl sulfate–polyacrylamide gel electrophoresis were transferred to polyvinylidene difluoride membranes before being blocked for 60 min with 5% (w/v) bovine serum albumin in Tris‐buffered saline with 0.1% (v/v) Tween 20 (TBST). Membranes were incubated overnight at 4°C with the following primary antibodies: phospho‐glycogen synthase (GS) (p‐GS, Ser641, 3891; Cell Signaling Technology). After incubation, the membranes were washed in TBST, incubated for 1 h at room temperature with secondary antibodies (A102PT, American Qualex), and washed again in TBST. Chemiluminescent reagents (RPN 2232 and RPN 2109; GE Healthcare Japan) were used for blot detection. The blots were scanned and quantified using ChemiDoc XRS (170‐8071; Bio‐Rad) and Quantity One software (170‐9600; Bio‐Rad).

### 2.6 Statistical analysis

All data are expressed as mean ± standard error of the mean. In experiments involving rectal temperature, glucose concentration, and insulin concentration, a two‐way repeated measures analysis of variance (ANOVA) was performed to examine the effects of time and treatment. For the other experiments, statistical analysis was performed using one‐way ANOVA. When differences were found to be significant, comparisons were made using the Tukey–Kramer post‐hoc test. Significant differences and trends were defined as *p* < 0.05 and *p* < 0.10, respectively.

## 3 RESULTS

### 3.1 Rectal temperature

**FIGURE 1:**
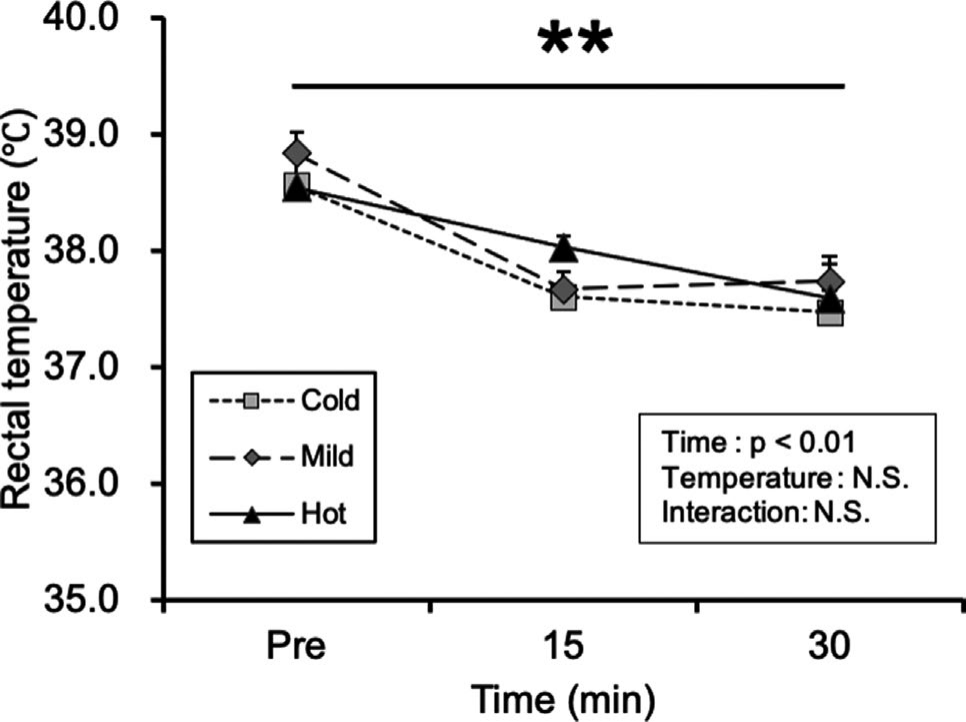
Rectal temperature. Values are presented as mean ± standard error of the mean with *n* = 6–8. **Main effect of time (*p* < 0.01). N.S., not significant; Cold, mice were administered with cold glucose solution (4°C); Mild, mice were administered with mild glucose solution (37°C); Hot, mice were administered with hot glucose solution (55°C); Pre, pre‐treatment

We first measured the rectal temperature to clarify the effect of solution temperature on core temperature. Rectal temperature decreased with the passage of time; however, there were no differences among the solution temperature groups (Figure 1; main effect of time: *p* < 0.01, temperature: not significant).

### 3.2 Blood substrate concentration

**FIGURE 2:**
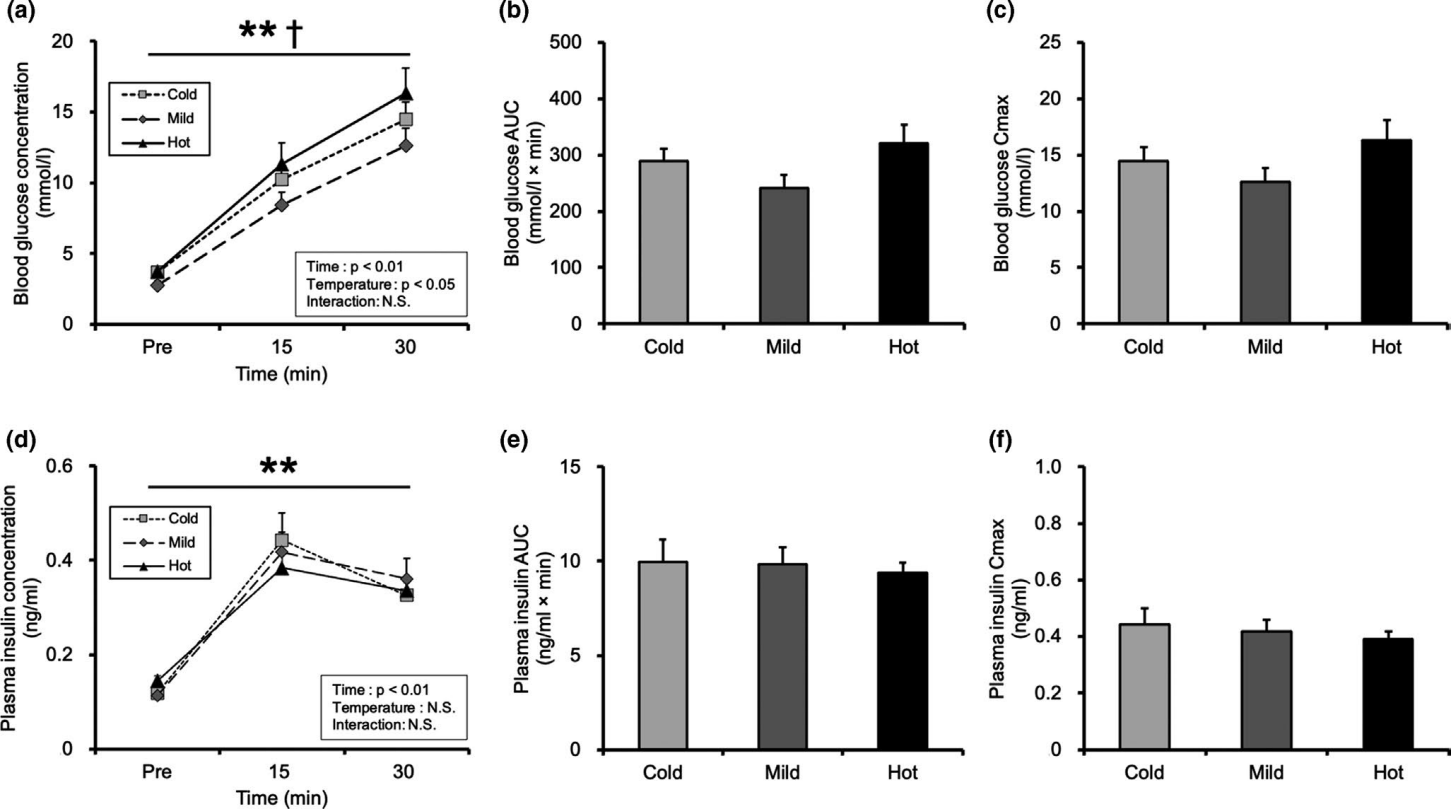
Blood and plasma substrate concentrations. (a) Blood glucose concentration. (b) Blood glucose area under the curve (AUC). (c) Maximum blood glucose concentration (Cmax). (d) Plasma insulin concentration. (e) Plasma insulin AUC. (f) Plasma insulin Cmax. Values are presented as mean ± standard error of the mean with *n* = 7–8. **Main effect of time (*p* < 0.01). †Main effect of temperature (*p* < 0.05). N.S., not significant; Cold, mice were administered with cold glucose solution (4°C); Mild, mice were administered with mild glucose solution (37°C); Hot, mice were administered with hot glucose solution (55°C); Pre, pre‐treatment

The blood glucose concentration increased with time (Figure 2a; main effect of time: *p* < 0.01). The main effect of temperature was observed for blood glucose concentration (Figure 2a; main effect of temperature: *p* < 0.05). However, the Tukey–Kramer multiple comparison test showed no significant difference in blood glucose concentrations among the three groups (Figure 2a). In addition, there were no differences in the blood glucose area under the curve (AUC) and maximum blood glucose concentration (Cmax) among the three groups (Figure 2b and c). The plasma insulin concentration increased with time; however, no differences were observed in solution temperature (Figure 2d; main effect of time: *p* < 0.01). The plasma insulin AUC and Cmax were similar among the three groups (Figure 2e and f).

### 3.3 Muscle glycogen concentration

**FIGURE 3:**
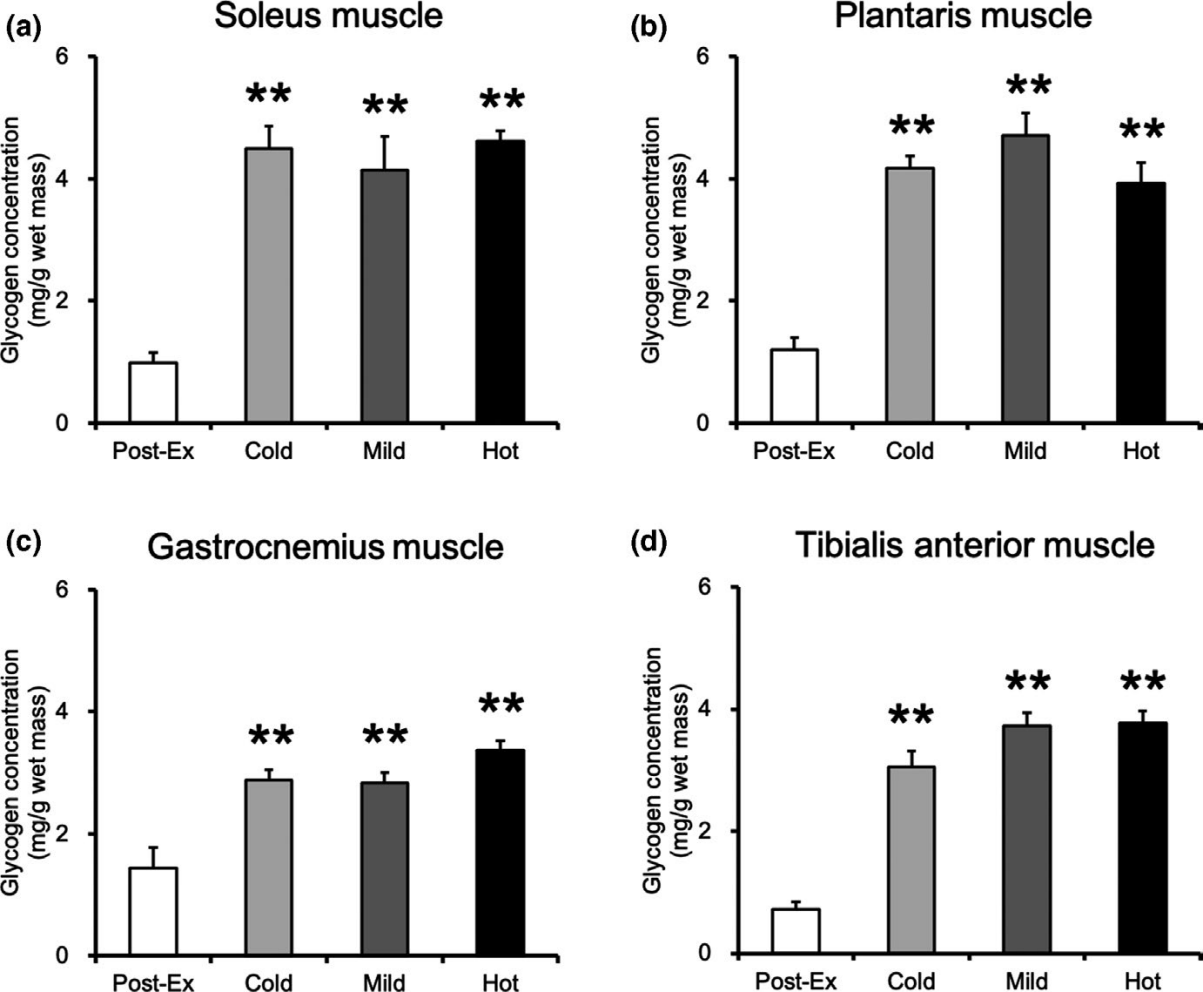
Glycogen concentration in skeletal muscles at 30 min after glucose ingestion. (a) Soleus. (b) Plantaris. (c) Gastrocnemius. (d) Tibialis anterior. Values are presented as mean ± standard error of the mean with *n* = 6–8. **Significantly different from the postexercise group (*p* < 0.01). Cold, mice were administered with cold glucose solution (4°C); Mild, mice were administered with mild glucose solution (37°C); Hot, mice were administered with hot glucose solution (55°C); Post‐Ex, post‐exercise

Glycogen concentrations (mg/g wet weight mass) in the muscles of the sedentary group were as follows: soleus, 5.5 ± 0.6; plantaris, 6.6 ± 0.3; gastrocnemius, 6.0 ± 0.2; and tibialis anterior, 6.7 ± 0.3. At 30 min after exercise, the glycogen content of the soleus, plantaris, gastrocnemius, and tibialis anterior muscle was significantly lower in the postexercise group than those in the three glucose ingestion groups (Figure 3a–d, *p* < 0.01). However, the muscle glycogen content did not differ among the three groups.

### 3.4 Liver glycogen concentration

**FIGURE 4:**
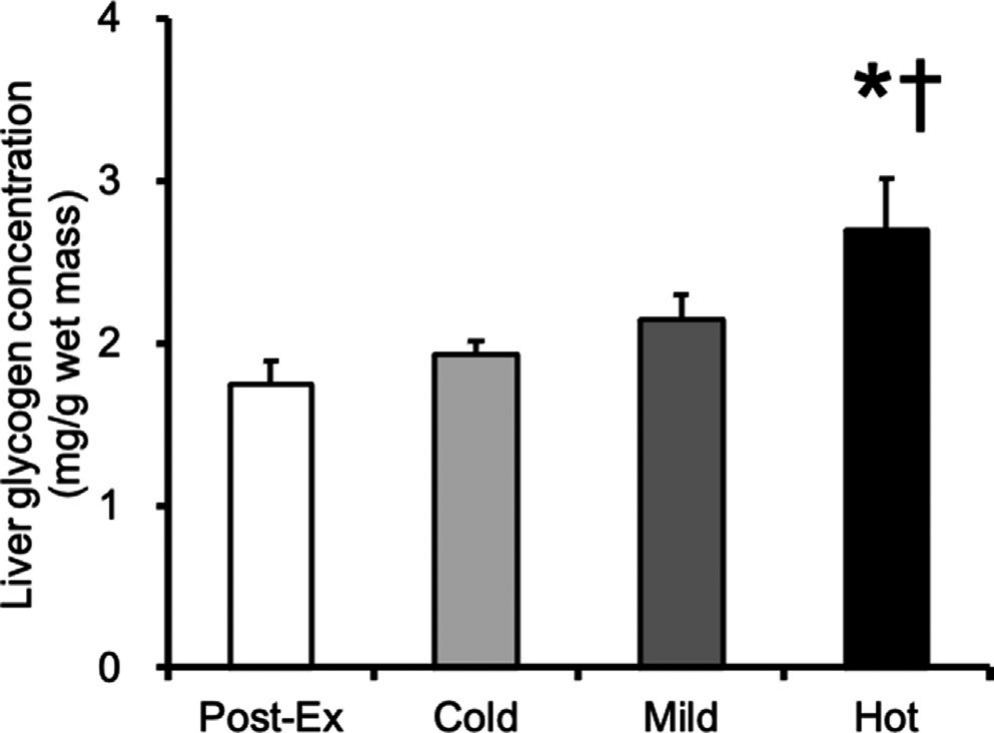
Glycogen content at 30 min after glucose ingestion in the liver. Values are presented as mean ± standard error of the mean with *n* = 6–8. *Significantly different from the postexercise group (*p* < 0.05). †Significantly different from the cold solution group (*p* < 0.05). Cold, mice were administered with cold glucose solution (4°C); Mild, mice were administered with mild glucose solution (37°C); Hot, mice were administered with hot glucose solution (55°C); Post‐Ex, post‐exercise

The liver glycogen concentration in the sedentary group was 51.6 ± 4.4 mg/g wet weight mass. At 30 min after exercise, the liver glycogen concentration in the Hot group was significantly higher than that in the post‐exercise and Cold groups (Figure 4, *p* < 0.05). The liver glycogen concentrations in the Cold and Mild groups were similar to those in the postexercise group.

### 3.5 Liver metabolites and glycogen synthase

**FIGURE 5:**
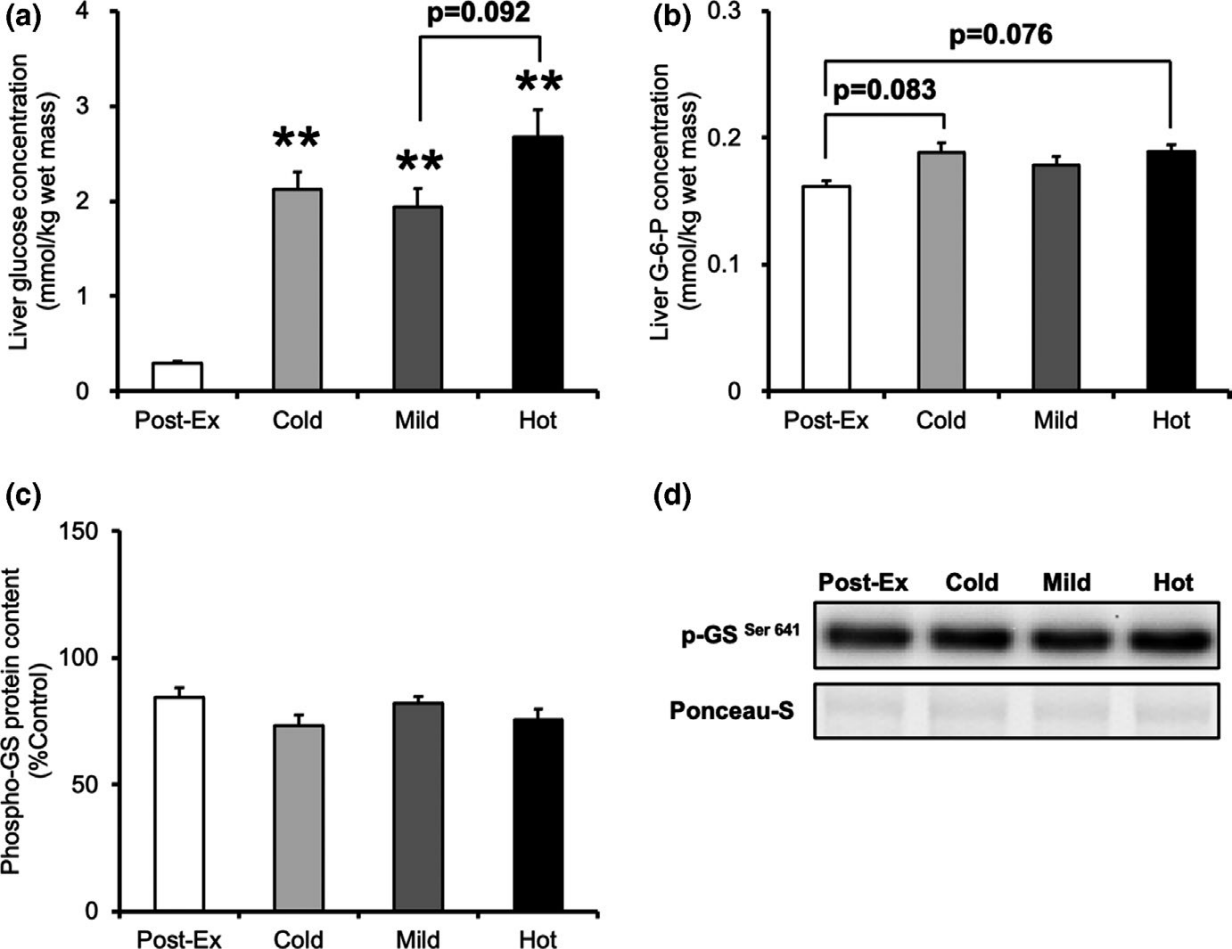
Tissue metabolite and phosphorylation protein levels in the liver at 30 min after glucose ingestion. (a) Liver glucose concentration. (b) Liver G‐6‐P concentration. (c) p‐ GS ^Ser641^ protein content. (d) Representative western blots for p‐GS ^Ser641^. Values are presented as mean ± standard error of the mean with *n* = 5–8. **Significantly different from the postexercise group (*p* < 0.01). Cold, mice were administered with cold glucose solution (4°C); Mild, mice were administered with mild glucose solution (37°C); Hot, mice were administered with hot glucose solution (55°C); Post‐Ex, post‐exercise; G‐6‐P, glucose‐6‐phosphate; p‐GS, phospho‐glycogen synthase

To clarify the mechanism of the effect of different solution temperatures on liver glycogen recovery, we measured liver glucose and G‐6‐P concentrations in addition to p‐GS protein content. The liver glucose concentrations in the three treatment groups were significantly higher than those in the postexercise group (*p* < 0.05). The liver glucose concentration in the Hot group tended to be higher than in the Mild group (Figure 5a, *p* = 0.092). The liver G‐6‐P concentrations in the Cold and Hot groups tended to be higher than those in the postexercise group (Figure 5b, *p* = 0.083 vs. *p* = 0.076). Further, the p‐GS protein concentration was similar among the groups (Figure 5c and d).

### 3.6 Intragastric and portal glucose concentration

**FIGURE 6:**
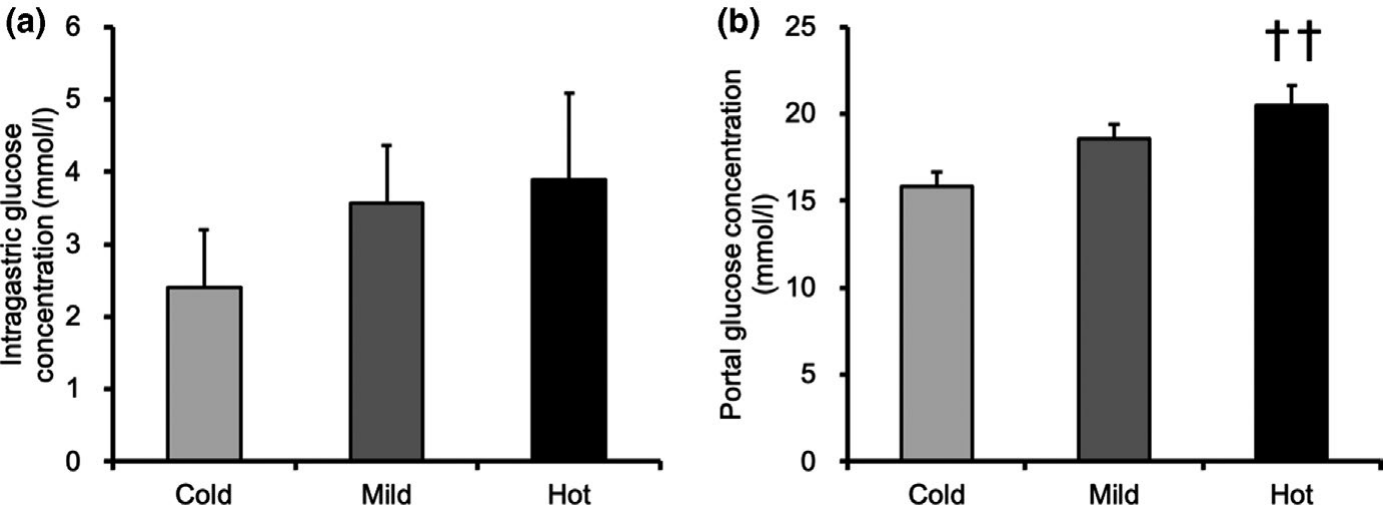
Intragastric and portal glucose concentrations. (a) Intragastric glucose concentration. (b) Portal glucose concentration. Values are presented as mean ± standard error of the mean with *n* = 6–7. ††Significantly different from the cold solution group (*p* < 0.01). Cold, mice were administered with cold glucose solution (4°C); Mild, mice were administered with mild glucose solution (37°C); Hot, mice were administered with hot glucose solution (55°C)

After absorption in the digestive tract, glucose is transported to the liver through the portal vein. Evaluation of intragastric and portal glucose concentrations revealed that intragastric glucose concentration increased in a temperature‐dependent manner, but the difference was not statistically significant (Figure 6a). The portal glucose concentration in the Hot group was significantly higher than that in the Cold group (Figure 6b, *p* < 0.01).

## 4 DISCUSSION

To the best of our knowledge, this is the first study to determine the effect of the temperature of different ingested carbohydrate solutions on glycogen recovery. Our results showed that postexercise muscle glycogen repletion did not differ with respect to the temperature of the ingested solution. Meanwhile, hot glucose solution ingestion enhanced postexercise liver glycogen recovery compared with cold glucose solution ingestion.

Since muscle glycogen is an energy substrate and a determinant of exercise capacity (Bergström et al., 1967; Skein et al., 2012), rapid restoration of muscle glycogen after exercise is vital. Glucose intake enhances muscle glycogen recovery (Pascoe et al., 1993). In this study, muscle glycogen concentrations were significantly higher in the Cold, Mild, and Hot groups than in the postexercise group, suggesting that muscle glycogen concentrations began to recover within 30 min of solution ingestion in all three groups. This result is consistent with a previous study (Conlee et al., 1978). However, there was no difference in any of the skeletal muscles among the three groups in this regard. During glycogen synthesis in the skeletal muscle, the uptake of blood glucose into the skeletal muscle is promoted by insulin (Zawadzki et al., 1992). Therefore, blood glucose and insulin concentrations play a major role in muscle glycogen synthesis. In this study, there was no difference in either glucose or insulin concentration among the three groups. Our result suggests that the temperature of the solution does not affect the recovery of muscle glycogen concentration. Furthermore, muscle glycogen recovery occurs preferentially to liver glycogen after exercise (Ivey & Gaesser, 1987). Thus, the muscle glycogen concentration recovers to a certain extent after ingestion of the glucose solution, regardless of its temperature.

Unlike the muscle results, liver glycogen recovery was greater after ingestion of the hot solution than that after ingestion of the cold solution in this study. In a previous study, 29.3% of the ingested glucose was initially extracted by splanchnic tissues (Meyer et al., 2002). The liver expresses glucose transporter 2 (GLUT2), which is responsible for glucose transport (Marín‐Juez et al., 2015). As GLUT2 has a low glucose affinity, an elevation in the postprandial blood glucose concentration increases the glucose transport rate and intracellular glucose concentration (Wu et al., 1998). Glucose is absorbed mainly in the small intestine and is delivered to the liver via the portal vein. Liver glucose uptake is higher when glucose is infused into the portal vein than that when it is infused into the periphery (Ishida et al., 1983), resulting in higher liver glycogen accumulation in portal vein infusion (Pagliassotti et al., 1996). Thus, increasing portal glucose concentration would enhance liver glucose uptake and glycogen repletion. In this study, the portal glucose concentration was higher in the Hot group than that in the Cold group. Liver glucose and G‐6‐P concentrations in the Hot group were marginally higher than those in the other groups. Furthermore, we set a fasting period of 16 hours in this study. Compared to the more typical physiological 5 h fasting period, an extended overnight period of fast is known to almost deplete liver glycogen content completely (Ayala et al., 2006). Hence, we inferred that more glucose was absorbed in the intestine and taken up by the liver in the Hot group at 30 min after exercise. Meanwhile, the reaction catalyzed by glycogen synthase, which is activated by dephosphorylation, is the rate‐limiting step in glycogen synthesis (Nuttall et al., 1988). In this study, there was no difference in the phosphorylation of glycogen synthase among the different groups. Therefore, our observation suggest that hot solution intake enhances glycogen repletion by promoting glucose uptake into the liver.

The liver, where glycogen is mainly stored as a glucose reservoir for other tissues (Bollen et al., 1998), is involved in the stabilization of blood glucose. As with muscle, glucose utilization in the liver increases during exercise and liver glycogenolysis increases in an exercise intensity‐dependent manner (Gonzalez et al., 2016). In fact, splanchnic glucose output increased approximately 2‐, 3‐, and 5‐fold after 40 min of bicycle ergometer exercise at work loads of 400, 800, and 1200 kg‐m/min, respectively (Wahren et al., 1971). Endurance exercise for 4 h reduces blood glucose levels by approximately 30% from basal levels (Ahlborg et al., 1974). These findings imply that if the intensity and duration of exercise are increased, liver glycogen may also be depleted and the energy supply to the muscle may not be successful. Therefore, enhancing liver glycogen recovery is important for replenishing energy substrates and supplying glucose to working muscles. In addition, postexercise glucose ingestion enhances glucose uptake in the liver compared with that in the sedentary control (Galassetti et al., 1999). This is believed to promote the liver glycogen recovery after exercise. In the present study, liver glycogen concentration in the Hot group was higher than that in the Cold group. This observation suggests that ingesting hot glucose solution after exercise can be an effective means for liver glycogen repletion compared with cold glucose solution ingestion.

Costill and Saltin (1974) reported that intake of a cold solution of glucose with NaCl (5°C) accelerated gastric emptying more than the intake of a mild solution (35°C). The present study showed that the glucose concentration in the stomach differed in a temperature‐dependent manner, although no statistical difference was observed. Thus, we could not deny the possibility that gastric emptying was faster and that the peak of absorption in the intestine had passed by the sampling time, resulting lower portal glucose in the Cold group. It is important to note, however, that blood glucose and muscle glycogen concentrations in the Cold group were not different from those in the other groups, and the liver glycogen concentration was lower than that in the Hot group. Consequently, it is possible that while the Cold group had faster gastric emptying, the glucose absorption did not catch up with gastric emptying, resulting in smaller amount of glucose absorption.

Because we expected that digestion and absorption would affect the early phase of postexercise glycogen recovery, we measured recovery in a short period of 30 min after administration. In addition to the enhanced liver glycogen repletion with hot glucose solution intake, the glucose concentration in portal blood, which supplies the energy substrate, was also high. Thus, it is possible that the hot glucose solution intake will enhance glycogen repletion even if the recovery time is extended. However, considering that liver glucose, G‐6‐P, and p‐GS were not statistically different among the three groups in this study, the liver glycogen may not be different when recovery time is extended. Thus, it is necessary to examine the key factors for liver glycogen recovery and the effects of solution temperature on longer recovery periods. Since it was difficult to control the estrous cycle of the mice in this study, we used only males. Because it has been reported that females exhibit similar muscle glycogen recovery compared to males (Flynn et al., 2020), we presume that hot glucose solution enhances glycogen repletion in female mice like male mice. However, females have also been reported to have lower respiratory exchange ratios and muscle glycogen utilization during exercise (Tarnopolsky et al. 1990), and reportedly stored lower levels of liver glycogen compared to males (Gustavsson et al., 2010). Additionally, females in the luteal phase reportedly had lower glycogen utilization during exercise compared with females in the follicular phase of the menstrual cycle (Devries et al., 2006). Therefore, the results of this study cannot be directly applied to females and need to be interpreted accordingly.

### 4.1 Limitations

Given that the temperature of the ingested solution would affect the amount of glucose absorption, we measured the portal blood and intragastric glucose concentrations. However, the amount of glucose absorption in the small intestine was not measured rigorously. It is necessary to examine the effects of solution temperature on the amount of glucose absorption in the small intestine. Moreover, it is possible that glycogen may be restored by water intake, although only slightly; therefore, the water intake group should be included in a future study.

## 5 CONCLUSION

The present study examined the effects of different solution temperatures of postexercise glucose ingestion on glycogen recovery. No difference in insulin secretion or muscle glycogen recovery was observed among the solution temperatures. In contrast, hot glucose solution ingestion enhanced the portal glucose concentration and liver glycogen recovery compared with cold glucose solution ingestion. These observations suggest that postexercise muscle glycogen repletion occurs regardless of glucose solution temperature, and that ingesting hot glucose solution after exercise can be an effective means for liver glycogen repletion. The results of this study are expected to add new insights to the literature regarding postexercise gastrointestinal absorption and liver glycogen synthesis; however, the detailed mechanism requires further investigation.

## DISCLOSURE STATEMENT

The authors declare no competing interests.

## AUTHOR CONTRIBUTIONS

Y.M., S.K., and H.H. conceived and designed the study. Y.M., S.K., Y.T., T.S., and H.Y. conducted the experiments. Y.M., S.K., and K.T. analyzed the data. Y.M., S.K., K.T., Y.T., and H. H. wrote the manuscript. All authors contributed to the interpretation of the data and manuscript preparation. All authors read and approved the final version of the manuscript.
